# Twelve Weeks of High-Intensity Interval Training Alters Adipose Tissue Gene Expression but Not Oxylipin Levels in People with Non-Alcoholic Fatty Liver Disease

**DOI:** 10.3390/ijms24108509

**Published:** 2023-05-09

**Authors:** Susanne Csader, Marsena Jasiel Ismaiah, Tiina Kuningas, Merja Heinäniemi, Janne Suhonen, Ville Männistö, Heikki Pentikäinen, Kai Savonen, Milla-Maria Tauriainen, Jean-Marie Galano, Jetty Chung-Yung Lee, Reeta Rintamäki, Piia Karisola, Hani El-Nezami, Ursula Schwab

**Affiliations:** 1 Department of Public Health and Clinical Nutrition, University of Eastern Finland, FI-70200 Kuopio, Finland; 2 School of Biological Sciences, The University of Hong Kong, Pokfulam Road, Hong Kong 999077, China; 3 Institute of Biomedicine, School of Medicine, University of Eastern Finland, FI-70210 Kuopio, Finland; 4 Department of Medicine, University of Eastern Finland and Kuopio University Hospital, FI-70210 Kuopio, Finland; 5 Kuopio Research Institute of Exercise Medicine, FI-70210 Kuopio, Finland; 6 Department of Clinical Physiology and Nuclear Medicine, Kuopio University Hospital, FI-70210 Kuopio, Finland; 7 Institut des Biomolécules Max Mousseron (IBMM), UMR 5247, Université de Montpellier, CNRS, ENSCM, F-34093 Montpellier, France; 8 Department of Medicine, Endocrinology and Clinical Nutrition, Kuopio University Hospital, FI-70210 Kuopio, Finland; 9 Faculty of Medicine, Human Microbiome Research Program, University of Helsinki, FI-00100 Helsinki, Finland

**Keywords:** non-alcoholic fatty liver disease, adipose tissue, exercise, gene expression, RNA, oxylipin, hemoglobin, human

## Abstract

Lifestyle modifications, including increased physical activity and exercise, are recommended for non-alcoholic fatty liver disease (NAFLD). Inflamed adipose tissue (AT) contributes to the progression and development of NAFLD and oxylipins such as hydroxyeicosatetraenoic acids (HETE), hydroxydocosahexanenoic acids (HDHA), prostaglandins (PEG_2_), and isoprostanoids (IsoP), which all may play a role in AT homeostasis and inflammation. To investigate the role of exercise without weight loss on AT and plasma oxylipin concentrations in NAFLD subjects, we conducted a 12-week randomized controlled exercise intervention. Plasma samples from 39 subjects and abdominal subcutaneous AT biopsy samples from 19 subjects were collected both at the beginning and the end of the exercise intervention. In the AT of women, a significant reduction of gene expression of hemoglobin subunits (*HBB*, *HBA1*, *HBA2*) was observed within the intervention group during the 12-week intervention. Their expression levels were negatively associated with VO_2_max and maxW. In addition, pathways involved in adipocyte morphology alterations significantly increased, whereas pathways in fat metabolism, branched-chain amino acids degradation, and oxidative phosphorylation were suppressed in the intervention group (*p* < 0.05). Compared to the control group, in the intervention group, the ribosome pathway was activated, but lysosome, oxidative phosphorylation, and pathways of AT modification were suppressed (*p* < 0.05). Most of the oxylipins (HETE, HDHA, PEG_2_, and IsoP) in plasma did not change during the intervention compared to the control group. 15-F_2t_-IsoP significantly increased in the intervention group compared to the control group (*p* = 0.014). However, this oxylipin could not be detected in all samples. Exercise intervention without weight loss may influence the AT morphology and fat metabolism at the gene expression level in female NAFLD subjects.

## 1. Introduction

Non-alcoholic fatty liver disease (NAFLD), encompassing not only simple steatosis but also steatohepatitis (NASH), fibrosis, and cirrhosis, is the most common liver disease worldwide, with an estimated global prevalence of 32.4% [1,2]. It is associated with an increased risk of developing hepatocellular carcinoma, extrahepatic cancers, and increased overall mortality [3,4,5]. Lifestyle changes, including increased physical activity and exercise, are recommended to prevent NAFLD [6]. Exercise improves insulin sensitivity and decreases intrahepatic lipid content in subjects with NAFLD [7]. HIIT without weight loss showed a reduction of intrahepatic lipid (IHL) content, and improvement in the liver enzymes alanine aminotransaminase (ALT) and aspartate aminotransaminase (AST) [8]. Furthermore, positive effects of exercise on lipid and glucose profiles were observed. NAFLD subjects had reduced triglyceride and cholesterol concentrations but increased high-density lipoprotein cholesterol concentrations and improvements in insulin sensitivity after exercise [9,10,11]. In addition, exercise may have a positive influence on metabolic health through adipose tissue (AT) remodeling [12]. Recently it has been shown that high-intensity interval training (HIIT) without weight loss changed the subcutaneous adipose tissue (SAT) morphology in adults with obesity, such as reduced adipocyte size, modification of the extracellular matrix (ECM), and increased capillarization [13]. Visceral fat but also SAT are associated with NAFLD [14], and dysfunctional adipose tissue (AT) is closely linked with NAFLD [15]. Increased lipolysis, macrophage infiltration, inflammation in AT, and altered circulating adipokine levels contribute to the development and progression of NAFLD [2,16,17]. Oxylipins, oxygenated polyunsaturated fatty acids, are also responsible for the homeostasis and inflammation in AT and could possibly take part in NAFLD progression [18,19].

Oxylipins are bioactive mediators with a wide range of biological functions, and many of them are still being elucidated for their roles. Oxylipins such as hydroxyeicosatetraenoic acids (HETE), hydroxydocosahexanenoic acids (HDHA), prostaglandins (PEG_2_), and isoprostanoids (IsoP) are formed by enzymatic and non-enzymatic peroxidation of polyunsaturated fatty acids (PUFA). To date, they were found to be involved in, for instance, inflammation, immune function, oxidative stress, tissue repair, and cardiovascular function, and can be altered in some diseases [20]. For example, increased levels of 5- and 11-HETE were highly positively associated with BMI and waist circumference [21]. Increased concentrations of proinflammatory oxylipins were found in people with metabolic syndrome compared to healthy controls and in people with NAFLD and type 2 diabetes [18,22,23]. Oxylipins are mobilized while performing exercise, and their role during acute and chronic exercise is an emerging topic in exercise science [24]. A recent exercise study suggested that increased levels of a linoleic acid derived oxylipin, which increases skeletal muscle fatty acid uptake, derived from brown AT [25]. On the other hand, a prolonged and intensive exercise session was related to the activation of pro-inflammatory oxylipins [26].

We conducted a 12-week randomized controlled high-intensity interval training (HIIT) intervention in NAFLD subjects, which revealed several changes in metabolites in AT, plasma, urine, and stool [27]. We hypothesized that exercise alters the SAT gene expression levels and decreases oxidative stress, benefitting NAFLD improvement. Here we report the effect of exercise on SAT at the gene level and oxylipin concentrations in NAFLD subjects.

## 2. Results

### 2.1. Transcriptomic Analysis in Adipose Tissue

The multi-dimensional scaling (MSD) plot from 19 subjects revealed two clusters based on gender (Supplementary Figure S1). It was decided to perform differential gene expression analysis focusing on women (n = 14), since there were seven women in both groups and the inter-individual expression level variation was lower.

The exercise parameters maximal oxygen consumption (VO_2_max) and maximal power (maxW) were significantly higher in the intervention group, compared to the control group. In addition, ALT was significantly elevated in the intervention group compared to the control group at the end of the study, which might be a result of exercise stress [28,29] (Table 1).

**Figure 1:**
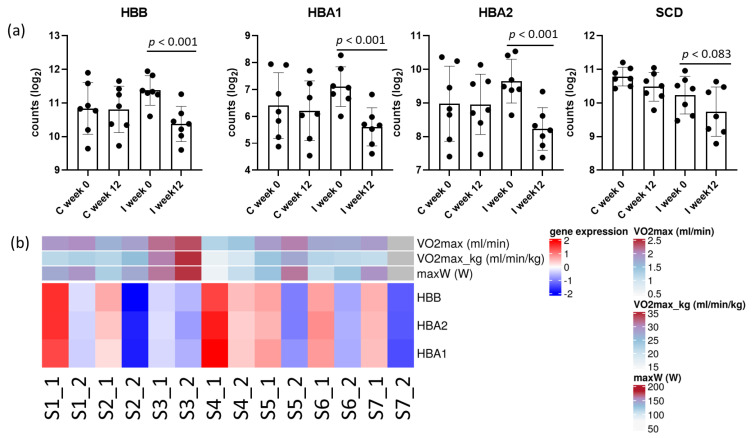
(**a**) Significantly differentially expressed genes (*HBB*, *HBA1*, *HBA2*) and a gene with a change approaching significance (*SCD*) in the intervention group in adipose tissue after the adjustment (FDR < 0.1); (**b**) associations between exercise parameters and expression levels of hemoglobin subunits in the intervention group. Expression levels are shown as z-scored values; S1_1, sample1 at baseline (week 0); S1_2, sample 1 at endpoint (week 12); no exercise data from S7_2 were obtained.

In total, DGE analysis revealed 316 genes with a raw *p*-value < 0.05 within the intervention group, and 537 genes between the control and intervention groups (Supplementary Table S1). After multiple hypothesis testing adjustment, at an estimated 10% false discovery rate (FDR), three hemoglobin encoding genes within the intervention group (*HBA1 p* < 0.001, *HBA2 p* < 0.001, and *HBB p* < 0.001) showed significant changes, and lipid metabolism gene stearoyl-CoA desaturase (*SCD p* = 0.083) tended to be significant during the study in the intervention group (Figure 1a). However, no gene remained significant between the intervention with the control group at the end of the study. *HBA1*, *HBA2*, and *HBB* were in the most samples to be negatively associated with VO_2_max and maxW (Figure 1b). There was a trend with decreased *SCD* expression and reduced fat mass and visceral fat area. However, the expression level variation between the samples was high (Supplementary Figure S2).

**Figure 2:**
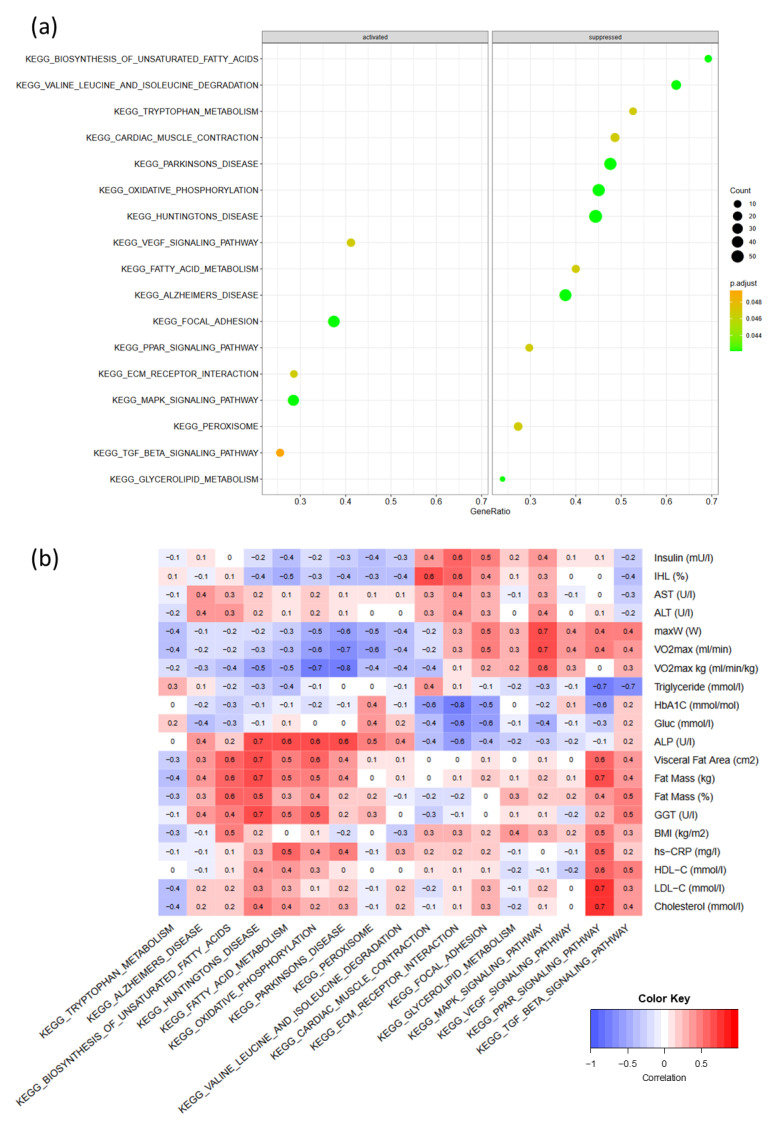
(**a**) Significant activated and suppressed pathways in adipose tissue within the intervention group in a 12-week HIIT; (**b**) Pearson’s correlation between significant pathways and clinical parameters; EMC, extracellular matrix; MAPK, mitogen-activated protein kinase; PPAR, peroxisome proliferator-activated receptor; TGF, transforming growth factor; VEGF, vascular endothelial growth factor; increased size of dots shows increased number of gene counts involved in the pathway; gene ratio is the ratio of significantly differentially expressed genes vs. all annotated genes per pathway; the color shows the *p*-values; the brighter the green, the lower the *p*-value.

Within the intervention group, the GSE analysis for genes ranked by significance showed 17 significant altered pathways using the KEGG database (Figure 2a). Among the significant pathways, genes belonging to pathways involved in AT remodeling and inflammation were activated, i.e., VEGF signaling (p_adj_ = 0.049), focal adhesion (p_adj_ = 0.036), EMC–receptor interaction (p_adj_ = 0.049), MAPK signaling (p_adj_ = 0.049), and TGF-beta signaling (p_adj_ = 0.049). In comparison, genes belonging to pathways of fatty acid metabolism, i.e., biosynthesis of unsaturated fatty acids (*p* = 0.036), fatty acid metabolism (p_adj_ = 0.049), PPAR signaling (p_adj_ = 0.036), glycerolipid metabolism (p_adj_ = 0.049), AA metabolism (valine, leucine, and isoleucine degradation) (p_adj_ = 0.036), and tryptophan metabolism (p_adj_ = 0.036), as well as oxidative phosphorylation (p_adj_ = 0.036) were suppressed (Figure 2a).

The correlation analysis between the pathway gene set score at the single sample level and clinical parameters showed only one significant negative correlation between HbA1c and the ECM–receptor interaction pathway (r = −0.8, *p* = 0.039). Strong negative (r = −0.7, *p* = 0.052) but non-significant correlations were seen between triglyceride concentrations and the PPAR signaling pathway. In addition, exercise parameters were strongly associated with the MAPK signaling pathway (r = 0.7, *p* = 0.150) and negatively associated with oxidative phosphorylation (r = −0.7, *p* = 0.064). Fat mass and visceral fat area were strongly and positively but non-significantly associated (r = 0.6, *p* = 0.198) with the biosynthesis of the unsaturated fatty acid pathway (Figure 2b).

**Figure 3:**
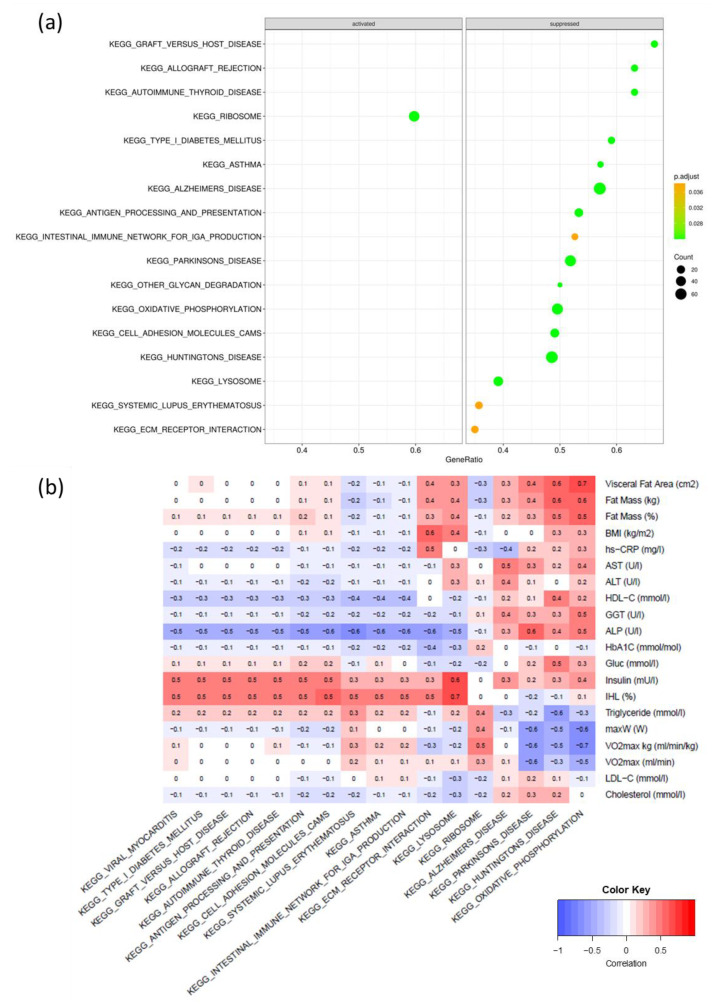
(**a**) significant pathways in adipose tissue between the intervention and control groups in a 12-week high-intensity interval training intervention; (**b**) Pearson’s correlation between significant pathways and clinical parameters; EMC, extracellular matrix; increased size of dots shows increased number of gene counts involved in the pathway; gene ratio is the ratio of significantly differentially expressed genes versus all annotated genes per pathway; the color shows the *p*-values; the brighter the green, the lower the *p*-value.

Between intervention and control, after the multiple hypothesis testing adjustment, 17 pathways were differentially expressed (Figure 3a). Out of the five pathways relevant to this study, the ribosome pathway (p_adj_ = 0.022) was activated and oxidative phosphorylation (p_adj_ = 0.021), lysosome (p_adj_ = 0.021), cell adhesion molecules cams (p_adj_ = 0.021), and ECM–receptor interaction (p_adj_ = 0.021) were suppressed in the intervention group compared to the control group as a result of the 12-week HIIT intervention. The correlation analysis revealed no significant negative correlation (Figure 3b).

### 2.2. Oxylipins in Plasma

Clinical data of the whole study group were previously reported [27]. In this study, there was no significant difference in the fatty acid profile between the control and intervention groups (Supplementary Table S2). A total of 16 oxylipins were measured in plasma in 38 subjects (male and female) at week 0 and week 12. Table 2 shows the normalized concentration at baseline and 12 weeks.

**Figure 4:**
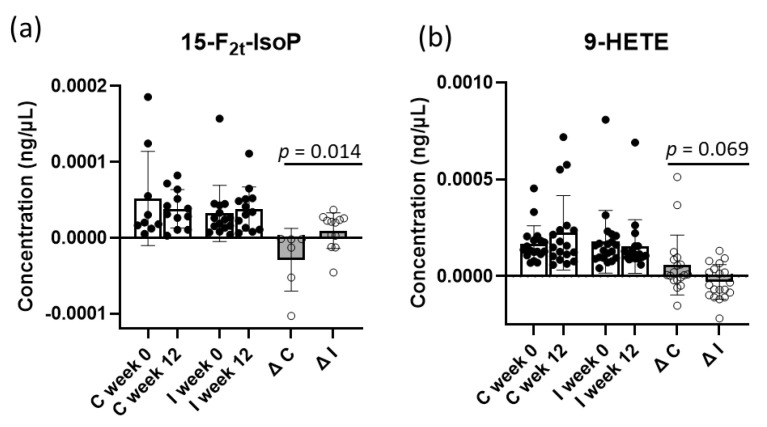
(**a**) 15-F_2t_-Isprostane (15-F_2t_-IsoP), (**b**) 9-hydroxyeicosatetraenoic acid (9-HETE), normalized with arachidonic acid concentrations of control (C) and intervention (I) groups at baseline and endpoint.

There were no significant differences between the groups in oxylipin concentrations at baseline, except for 12-HETE, which was lower in the intervention group compared to the control group (*p* = 0.028). At the end of the intervention, 15-F_2t_-IsoP increased significantly in the intervention group compared to the control group, in which the concentration decreased at the end of the study (*p* = 0.014). However, 15-F_2t_-IsoP could only be found in 15 subjects (Figure 4a). 9-HETE, which was slightly lower in the intervention group but higher in the control group, tended to be more significant compared to the control group (*p* = 0.069) (Figure 4a,b). Dividing the data according to gender, no significant changes between intervention and control group in men and women were observed.

## 3. Discussion

This thoroughly conducted 12-week randomized, controlled exercise intervention study with supervised exercise sessions demonstrated significant changes in SAT gene expression levels influencing adipocyte morphology and the fat metabolism in AT but no changes in plasma oxylipin concentrations in subjects with NAFLD.

### 3.1. AT Transcriptomics

The gene expression levels of hemoglobin (HB) subunits (*HBB*, *HBA1*, and *HBA2*) decreased significantly during the 12-week HIIT intervention in the female intervention group. Previous studies have shown that hemoglobin levels in the blood were associated with exercise performance [30,31], and a recent study has shown that jogging could improve plasma hemoglobin levels [32]. However, we saw a negative association between lower hemoglobin gene expression levels and increased fitness parameters. These contradictory results might be due to differing study subjects and exercise regimes. The above-mentioned studies were either conducted only in men [30] or resistance training was performed [31]. Higher plasma hemoglobin concentrations are associated with insulin resistance, hyperinsulinemia, and higher total cholesterol and triglycerides, as well as LDL cholesterol concentrations, blood pressure, metabolic syndrome, and higher mortality [33,34,35,36,37]. In addition, recently published studies showed an association between high Hb concentrations and high concentrations of ALT and the risk of fatty liver [37]. A possible explanation could be the induction of the hypoxia inducible factor (HIF), which leads to beneficial metabolic reprogramming [38]. However, other studies showed lower Hb concentrations in subjects with genetically-determined higher BMI and lipid metabolism [39], and hyperlipidemia was linked with the risk of anemia [40]. Our results could indicate a beneficial effect of decreased Hb levels in women with NAFLD performing aerobic exercise. However, the plasma Hb concentrations were not measured and also the HIF gene expression did not significantly change during the intervention.

The GSE analysis in SAT revealed some significant pathways within the exercise intervention group and between the intervention and control groups as a result of the 12-week HIIT intervention after adjusting the *p*-values. However, the genes within the pathways were non-significant after the adjustment. This could be due to the limited number of subjects and their high inter-variability of the gene expression levels.

Pathways involved in AT remodeling, such as the activated pathways of focal adhesion, EMC–receptor interaction, and VEGF due to 12 weeks of HIIT, were noticed. Additionally, a 12-week HIIT study in overweight/obese subjects without weight loss showed differences in EMC and increased angiogenesis in SAT after the intervention [13]. In our study, we found higher RNA expression levels of collagen type VI *Col6A3.* This fibrillar collagen was associated with insulin resistance and inflammation in AT [41,42]. However, compared to the control group, these collagen fibers did not change in the 12 weeks of the study. In addition, the expression level of thrombospondin1 (*THBS1*) found in focal adhesion, EMC– receptor interaction, and the TGF-beta pathway was higher within the exercise group. This glycoprotein was previously associated with BMI, hyperglycemia, and hypertension and noted as a risk factor for NAFLD in obese children [43,44]. Together with the activated pathway of TGF-beta and MAPK signaling, exercise might increase the inflammation in SAT in NAFLD subjects, which was also found in the 12-week HIIT study without weight loss [13].

Other genes in the ECM interaction pathway, which decreased overall, were reduced in the intervention group compared to the control group within the 12 weeks. Osteopontin, an ECM glycoprotein involved in tissue remodeling and inflammatory processes, was found to be highly expressed in the AT of obese people [45]. Moreover, osteopontin gene levels (*SPP1*) were significantly lower in our intervention group compared to the control group. A study has shown reduced macrophage infiltration in AT and improved insulin resistance in osteopontin-deficient mice [46], suggesting that decreased expression of osteopontin might alleviate inflammation in AT. Furthermore, suppressed expression of several genes in the cell adhesion molecule pathway was observed—for example, a significant reduction in genes of major histocompatibility complex II (MHCII). MHCII antigen representation in adipocytes increased in mice after feeding them a high-fat diet for two weeks, leading to increased proinflammatory T-cell activation [47]. A decrease in MHCII expression levels might implicate a decrease in T1 macrophage infiltration in AT. This is further supported by downregulated EMC proteoglycans, versican (*VCAN*), and biglycan (*BGN*) in the intervention group compared to the control group. These proteoglycans, vesican derived from adipocytes, and biglycan produced by macrophages are involved in angiogenesis, inflammation, and differentiations in cells and tissues [48,49]. Deletion of these proteoglycans leads to a reduction of macrophage accumulation and cytokine expression. In addition, adipocyte-specific deletion of versican decreased liver inflammation and increased glucose sensitivity [49]. Overall, HIIT seems to influence adipocyte structure and inflammation. However, its functional role is not yet clear.

In this study, the oxidative phosphorylation (OXPHOS) was lower in the intervention group when compared to the control group and negatively correlated with increased exercise parameters after HIIT. Several studies found an increase in OXPHOS as a result of exercise [50,51]. Additionally, no changes of OXPHOS were found in AT, but they were in muscle tissue, which might be due to different study setups [52]. Mitochondrial respiration was found to be lower in visceral fat but not in the SAT of obese people with NAFLD [53]. A recently published study showed increased mitochondrial respiration in SAT in insulin-resistant non-diabetic obese people compared to insulin-sensitive non-diabetic obese people [54]. The study’s authors assumed that the increased mitochondrial respiration could be a compensation mechanism to cope with the increased fatty acid (FA) spill over [54]. Based on these data, our observed decreased OXPHOS pathway might result from changes in the amount and composition of fatty acids within the adipocyte.

Within the intervention group, we observed suppressed genes of FA metabolism and biosynthesis of FA, suggesting that HIIT training alters FA composition in adipocytes. The stearoyl-CoA desaturase encoded by *SCD*, responsible for the transformation of saturated fatty acids (SFA) to monounsaturated fatty acids (MUFA), was suppressed within the intervention group in 12 weeks. Studies have shown that *SCD*-deficient mice showed reduced adiposity and adipocyte inflammation [55], and *SCD* activity was positively associated with obesity and insulin resistance in humans [56]. In addition, we saw a positive trend between decreased *SCD* expression and decreased fat mass and visceral fat area. While one study showed no differences in *SCD* activity as a result of exercise [57], exercise was assumed to reduce *SCD* activity by a changed ratio of MUFA and SFA [58], as well as lower *SCD1* levels in a recent mice study [59], which is in line with our results. One study showed increased triglyceride (TG) concentrations in overexpressed *SCD1* cells [60], indicating the TG production through endogenous synthesis of MUFA. Furthermore, another fatty acid desaturase, the delta-5 desaturase (*FADS1*), was significantly reduced in the intervention group as well. This enzyme is necessary for forming AA from linolic acid and EPA from alpha-linolenic acid.

In the exercise group, the gene diacylglycerol acyltransferase 2 (*DGAT2*), involved in glycerol lipid metabolism, was significantly lower in the exercise group. This gene is responsible for converting diacylglycerol into TG, whereby the incorporated FA might derive from de novo synthesized FA [61]. *DGAT2* is also associated with *SCD1*, and overexpressed *SCD1* and *DGAT2* cells resulted in higher TG concentrations than overexpressed cells with one of these two genes alone [60]. This could suggest that a reduction of *SCD1* and *DGAT2* leads to lower TG concentrations and smaller adipocyte sizes.

The transcriptomics analysis in SAT was only carried out in female subjects. It has to be noted that lipid and lipoprotein metabolism has major differences between females and males and is age-dependent [62,63]. The mechanisms of the sex differences in lipid metabolism are complex and often sex-hormone dependent (e.g., estrogen and androgen) [64]. For example, estrogens control the liver lipid metabolism. Women secrete very low-density lipoproteins with more integrated triglycerides, preventing the liver from fat accumulation [65]. A loss or decrease of estrogen leads to fat accumulation in animal experiments [66]. Additionally, women who had to undergo surgical operations inducing menopause had a two-fold higher risk of NAFLD [67]. In addition, men have a higher prevalence of NAFLD than reproductive women, but this sex difference is reduced or even reversed when comparing prevalence in menopausal women and men [68].

Overall, transcriptomic analysis revealed the dynamics of SAT in females in response to exercise. Changes in morphology and fat metabolism of adipocytes were observed. However, the plasma FA profile of the subjects was not modified in our study.

### 3.2. Plasma Oxylipins

Oxylipins are involved in many physiological processes, such as regulating immune and cardiac functions, as well as inflammation [24]. Exercise is shown to influence the production of oxylipins [20,24]. In our study, the concentration of 15-F_2t_-IsoP differed between the intervention and control groups in the 12-week exercise intervention. Isoprostanes, including 15-F_2t_-IsoP, are derived from arachidonic acid via non-enzymatic free radical induced peroxidation, which is now regarded as a specific biomarker for oxidative stress [69,70]. It is biologically a vasoconstrictor, regulating platelet activity, promoting arteriosclerosis, and inhibiting angiogenesis [71]. Gracia-Flores et al. (2018) demonstrated decreased 15-F_2t_-IsoP in the urine of athletes after exercise, suggesting exercise has beneficial effects on oxidative stress [72]. We noticed a significant increase of 15-F_2t_-IsoP in plasma at the end of the intervention. This is in line with several other studies showing increased levels of F_2_-IsoP in plasma after acute exercise [73]. The increase in this oxylipin could be a reaction and adaptation to exercise. It should be noted that 15-F2t-IsoP was only detected in 15 subjects. The rationale for this observation is unclear but may be due to inefficient hydrolysis of IsoP bound to phospholipid due to NAFLD [74]. This may also explain the limited number of oxylipins that were measurable in the plasma samples in this study.

Although not significant, 9-HETE showed a trend towards significance. While this oxylipin decreased in the intervention group, it increased in the control group. HETEs are arachidonic acid metabolites, oxidized by LOX. HETEs including 9-HETE are increased in obese NAFLD and NASH patients [75]. While one study did not find a significant change in 9-HETE [76], other studies observed increased levels of 9-HETE after acute exercise [77,78]. These different results could be due to different study designs. While the increased levels of 9-HETE were found in a singular exercise session [77], our results could imply an adaptation of exercise after a longer period or repeated exercise.

Fifteen of the studied oxylipins did not change significantly during our exercise intervention, although other studies found changes in these oxylipins after acute exercise [20,24]. The reasons for these different results are diverse. First, while acute exercise induces several changes in oxylipins, our observed results could mean an adaptation to exercise in 12 weeks. Another reason could be the storage time, which could influence oxylipin concentrations. One study showed no changes in oxylipin patterns stored at −80 °C for 15 months [79], but our storage time was around 20–24 months, which might influence the oxidized lipid concentrations. Another factor may be blood sampling time after the last exercise test. It has been shown that some oxylipin reaches pre-exercise concentrations after a five-hour recovery [20]. The maximum blood sampling time in this study was up to 24 h after the last exercise test, which might have had an influence on the oxylipin levels. Finally, the metabolite levels showed variation between individuals and the sample size was possibly too small to detect significant changes. More extensive studies are needed to explore the effect of oxylipins on metabolism during exercise.

Although this study was carefully conducted, it has some limitations. First of all, the sample size for transcriptome analysis is small. Due to the COVID-19 pandemic, we were unable to collect any more AT samples. Larger studies are warranted to verify our results. Furthermore, we included only female subjects for transcriptome analysis. As discussed above, lipid metabolism is gender-dependent. Therefore, it is important to carry out such studies also with male subjects. The results of transcriptomics cannot be transformed one-to-one into proteomics. Further studies analyzing the transcriptome as well as the proteome would be important in order to obtain a clear idea of the changes in AT due to exercise. The lipidomics analysis was performed in plasma only. It would be interesting to analyze it in AT as well. Furthermore, larger studies would be needed to study oxylipin, as its concentrations vary quite widely.

### 3.3. Perspectives

NAFLD is the most common liver disease worldwide and can lead to serious liver damage. This randomized controlled exercise study showed that HIIT without weight loss seems to remodel the adipocyte structure as well as the fat metabolism at gene level in female NAFLD subjects. Further investigations with larger cohorts could give us a better understanding of the role and mechanism of AT remodeling within the context of the adipose tissue–liver axis and the possible related changes in the liver during exercise. In addition, the study of oxylipins in exercise science is an emerging field. This study provided an overview of several oxylipins in male and female NAFLD subjects performing HIIT. Future studies may show the impact of oxylipin levels during exercise on NAFLD progression.

## 4. Materials and Methods

Plasma samples and AT samples were taken at the beginning and the end of a 12-week BestTreat HIIT intervention. The study protocol has been described previously [27]. Briefly, 46 subjects with NAFLD diagnosis were randomly assigned to an intervention group or a control group. The intervention group performed a supervised HIIT twice per week plus non-supervised low to moderate aerobic exercises once per week (e.g., walking or swimming) to achieve a total amount of the recommended 180 min of exercise per week [80]. The control group kept their physical activity unchanged during the study. Food records at weeks 0 and 12 were collected and checked by a clinical nutritionist. All subjects kept their diet unchanged [27]. In total, 39 subjects were included in the oxylipin analysis in plasma. Abdominal SAT biopsies were collected from the first 19 participants (14 women and 5 men). Due to the national COVID-19 regulations of people clustering, we could not collect the biopsies from the last 20 participants. The intrahepatic lipid content was measured by MRI.

Each subject in the intervention and control groups performed an ergospirometry test at baseline and at week 12. Based on the results at baseline, an individual training plan was tailored for the intervention group. The exercise protocol has been described before and was carried out on a cycle ergometer [27]. Briefly, the subjects performed HIIT sessions with a 5 min warm-up (30% of the hypothetical workload sustainable for 4 min (maxW_4_)) followed by five repeated bouts of 2–4 min high-intensity intervals (85% of maxW_4_) interspersed by 3 min of active recovery (20% of maxW4). The HIIT session ended with a 5 min cool down (20% of maxW_4_). All HIIT sessions were supervised and carried out twice per week for 12 weeks.

### 4.1. Transcriptomics in AT

Subcutaneous samples were taken via open biopsies, washed, and directly flash-frozen, as previously described (27).

For RNA extraction in AT, the RNeasy kit (Qiagen GMBH, Hilden, Germany) was used. First, 700 µLTriazole was added to 250–300 µg frozen AT and homogenized for 40 s with steel beads (TissueLyser LT, Qiagen, Germany). After the lysate was incubated for 5 min at room temperature, 140 µL chloroform was added, vortexed for 15 s, and incubated for 2–3 min at room temperature. The mixture was centrifuged at 12,000× *g* at 4 °C for 15 min. The aqueous supernatant was transferred to a new tube, and 1.5 volume of 100% ethanol was added and mixed with a pipette several times. A total of 700 µL of the mixture was added to the provided column and centrifuged at 12,000× *g* for 15 s. The flow-through was discarded. Afterwards, 700 µL of RWT buffer was added for a 15 s centrifugation, and the flow-through was discarded. Then, 500 µL of RPE buffer was added, and the column was centrifuged for 15 s. This step was repeated once more, but this time centrifuged for 2 min. To dry the column, it was further spun for 1 min. A volume of 30 µL RNAse-free water was directly pipetted onto the membrane and centrifuged for 1 min. The flow-through was used again to elute the RNA. The extracted RNA (RIN values ranged from 7.2 to 9.1, except one sample that had 6.0) was kept frozen at −80 °C until the analysis.

RNA sequencing was conducted using the Drop-seq method at the University of Helsinki [81]. The Nextera XT DNA sample prep kit (Illumina, Inc., San Diego, CA, USA) was used to prepare the library according to the manufacturer’s instructions, and the 3’end-amplified fragments were sequenced on the Illumina NextSeq 500 platform. Filtered and trimmed sequence reads shorter than 20 nt were trimmed with the help of the Trimmomatic (parameters: LEADING:3, TRAILING:3, SLIDING WINDOW: 4:15 and MINLEN:36). PolyA tails of a length of six or greater were removed by the Drop-seq tools (https://github.com/broadinstitute/Drop-seq, 31 March 2021). Then, the obtained sequences were mapped to the GRCm38.p6 whole genome using STAR (v2.6.0a, MIT, MA, USA) with the default settings for gene annotation. FeatureCounts software (v1.6.4, UniMelb, Melbourne, Australia) was used to calculate raw read counts. The mapping was performed on STAR.

### 4.2. Lipid Analysis

For fatty acid composition, collected plasma samples were analyzed with gas chromatography-mass spectrometry as fatty acid methyl esters (FAMEs). Details of the method are to be found in Supplementary Methods S3a.

### 4.3. Targeted Oxylipins Analysis

Folch extraction was used to extract the plasma lipids. After thawing the samples on ice, 10 μL of 1% BHT (dissolved in MeOH) was added to 1 mL of plasma samples. The mixture was centrifuged at 3000× *g* for 10 min at 4 °C to remove insoluble precipitates. A volume of 100 μL plasma was added to 5 mL of cold Folch solution (chloroform: MeOH, 2:1 *v*/*v* with 0.01% BHT), vortexed until a milky suspension appeared, and incubated on ice on an orbital shaker for 30 min. Afterwards, the mixture was centrifuged at 3100 rpm for 10 min. The supernatant was transferred to a new falcon tube and topped up with 5 mL of fresh cold Folch solution. To separate the phase, 2 mL of 0.9% NaCl was added and vortexed for 1 min. After incubation for 30 min on ice on an orbital shaker, the sample was centrifuged at 3100 rpm for 10 min, and the resulting lower phase was transferred to a 30 mL glass bottle. To the remaining upper layer, 2 mL chloroform was added, vortexed, and the lower phase was again transferred to the glass bottle. The last step was repeated. The solution was completely dried under nitrogen at 37 °C.

The lipid extracts were re-dissolved with 1 mL of 1 N KOH (in MeOH, with 0.01% BHT) plus 1 mL PBS (pH 7.4) and purged with nitrogen. The solution was then incubated overnight without light exposure. To stop the hydrolysis, 500 μL of 1N HCl, 0.5 mL 100% MeOH, 2.7 mL 40 mM formic acid, and 4 mL 20 mM formic acid were added. Finally, 100 μL of internal standard cocktail (0.1 ng/μL) was added. The hydrolyzed lipid was cleaned and extracted using solid phase extraction (SPE, Oasis). Subsequently, the SPE cartridges were washed with 2 mL of MeOH and next with 2 mL of 20 mM formic acid (pH 4.5). Then, the samples were loaded and washed first with 2 mL 2% NH_4_OH (*w*/*v*) and then with 20 mM formic acid. After that, the oxylipins were eluted with 6 mL hexane/EtOH/acetic acid (70:29.4:0.6, *v*/*v*/*v*).

The elute was dried under nitrogen at 37 °C until 0.5–1 mL of the elute remained, transferred to a new sample vial, and then dried until the end. The extract was re-suspended with 50 μL pure MeOH, filtered with a 0.45 μM PTFE syringe filter, and immediately analyzed using a liquid chromatography quadruple time-of-flight mass spectrometer (X500R QTOF system, Sciex Applied Biosystems, Framingham, MA, USA) consisting of an Exion LC AC liquid chromatograph with a C18 column maintained at 40 °C (150 × 2.1 mm, 2.6 μm particle size, Phenomenex, Torrance, CA, USA). Oxylipin (HETE, HDHA, and isoprostanoids) concentrations were normalized with the measured respective appropriate fatty acid measured as fatty acid methyl esters (FAME). The mass ions and the ionizing energy of the metabolites measured by the LC-MS/MS are shown in Supplementary Table S3.

### 4.4. Statistical Analysis

*Clinical parameters:* For intragroup comparisons, Wilcoxon signed rank tests were used and for comparing intergroup changes during the intervention, and Mann–Whitney‘s U test was performed.

*Transcriptomics analysis:* Differential gene expression (DGE) analysis was performed using R packages edgeR v. 3.26.8 [82] and limma v. 3.40.6 [83]. The protocol of Law et al. (2018), the third version, was used [84]. Gene set enrichment (GSE) analysis for comparisons between groups (intervention vs. control; end point vs. baseline) was conducted using the GSEA function from the clusterProfiler v3.12.0 R package [85]) with KEGG gene set collection “c2.cp.kegg.v2022.1.Hs.symbols.gmt” that was retrieved from MSigDB [86]. The log10 transformed *p* values from the DGE analysis (values of the downregulated genes, e.g., genes that had FC < 0 were multiplied by −1 to separate up- and downregulated genes) were used as the ranking metric. Dotplots were generated using the dotplot function in DOSE v. 3.10.2 R package [87]. To score gene set levels in individual samples, the ssgsea method from the gsva R package (1.42.0) was used [88]. Pearson’s correlation between pathway gene set scores and clinical parameters were calculated using the “rcorr” function from the “Hmisc” R-package. *p*-values were adjusted using the Benjamini and Hochberg method and the heatmap was plotted from the “gplots” R-package. The software R (version 4.2.2) was used for all analyses.

*Lipidomics analysis:* Oxylipins were normalized with the precursor arachidonic acid (AA) and docosahexaenoic acid (DHA). Mann–Whitney’s U-test was used for comparing the oxylipin concentrations (normalized with its precursor) between the intervention and control groups using the fold change. The analysis was performed using the software GraphPad Prism (version 9.2.0).

For all analyses, *p*-values  <  0.05 were considered statistically significant.

## Figures and Tables

**Table 1:** Clinical characteristics of 14 female subjects.

	Intervention (n = 7)		*p*-Value ^1^	Control (n = 7)		*p*-Value ^1^	*p*-Value ^2^
	W0	W12		W0	W12		
**T2D**	1			0			
**Age, years**	56.9 ± 12.2			61.3 ± 7.1			
**BMI, kg/m^2^**	29.3 ± 1.3	29.0 ± 1.6	0.310	31.1 ± 3.5	31.3 ± 3.4	0.310	0.225
**Fat mFass, kg**	29.4 ± 4.6	29.3 ± 5.2	0.553	34.9 ± 7.3	35.9 ± 7.2	**0.034**	0.085
**Fat mass, %**	37.5 ± 3.1	37.7 ± 3.6	0.885	40.8 ± 6.0	41.7 ± 6.1	**0.046**	0.159
**Visceral fat area, cm^2^**	145 ± 29	145 ± 32	0.310	177 ± 43	181 ± 45	0.176	0.338
**IHL, %**	11.24 ± 9.93	11.38 ± 8.81	0.735	17.59 ± 12.69	17.66 ± 11.11	0.866	0.886
**ALT, U/L**	44.14 ± 20.47	52.14 ± 17.06	0.248	55.00 ± 25.27	46.29 ± 18.92	**0.028**	**0.025**
**AST, U/L**	31.71 ± 7.18	35.29 ± 11.77	0.350	38.43 ± 12.93	35.29 ± 14.29	0.051	0.062
**ALP, U/L**	80.29 ± 28.38	87.71 ± 36.40	0.307	78.57 ± 21.17	76.14 ± 21.98	0.446	0.178
**GGT, U/L**	105.00 ± 91.11	133.71 ± 144.26	0.237	104.14 ± 118.31	97.43 ± 130.90	0.237	0.125
**Cholesterol, mmol/L**	5.34 ± 0.93	5.37 ± 1.18	0.916	4.91 ± 0.57	4.66 ± 0.56	0.108	0.199
**HDL-C, mmol/L**	1.48 ± 0.29	1.51 ± 0.46	0.397	1.64 ± 0.44	1.54 ± 0.45	0.051	0.141
**LDL-C, mmol/L**	3.64 ± 0.92	3.51 ± 0.99	0.408	3.06 ± 0.74	2.79 ± 0.65	0.058	0.438
**TG, mmol/L**	1.73 ± 0.57	1.67 ± 0.38	0.612	1.55 ± 0.53	1.56 ± 0.53	0.999	0.654
**Gluc, mmol/L**	6.0 ± 0.6	6.0 ± 0.5	0.595	6.0 ± 0.7	6.1 ± 0.6	0.167	0.248
**Insulin, mU/L**	13.57 ± 5.2	14.73 ± 5.63	0.235	23.26 ± 13.22	24.46 ± 13.20	0.735	0.654
**HbA1c, mmol/mol**	37.0 ± 5.7	38.1 ± 4.8	0.223	37.7 ± 3.0	38.9 ± 2.0	0.302	0.999
**hs-CRP, mg/L**	1.36 ± 0.75	1.75 ± 1.05	0.206	2.63 ± 1.14	2.33 ± 1.67	0.599	0.305
**VO_2_max, mL/min**	1.76 ± 0.31	1.88 ± 0.31	**0.046**	1.86 ± 0.23	1.85 ± 0.24	0.735	**0.031**
**VO_2_max, mL/min/kg**	23.02 ± 4.43	25.14 ± 5.31	**0.046**	22.09 ± 3.68	21.61 ± 4.01	0.866	**0.032**
**maxW, watt**	124.50 ± 25.38	144.17 ± 23.88	**0.027**	127.57 ± 25.16	124.57 ± 25.95	0.343	**0.004**

ALP, alaline phosphatase; ALT, alanine transaminase; AST, aspartate transaminase; BMI, body mass index; GGT, gamma-glutamyltransferase; Gluc, glucose; HbA1c, glycated hemoglobin; HDL-C, high-density lipoprotein–cholesterol; hs-CRP, heat shock C reactive protein; IHL, intrahepatic lipid content; LDL-C, low-density lipoprotein–cholesterol; T2D, type 2 diabetes; TG, triglyceride; VO2max, maximal oxygen consumption; maxW, maximal power; 1—Wilcoxon signed rank test; 2—*p*-values comparing fold changes of C and I during 12 weeks using Mann–Whitney’s U-test; bold marks significant values.

**Table 2:** Normalized oxylipin concentration in plasma (pg/mL) at baseline and endpoint (mean ± SD).

	Intervention (n = 20) Week 0	Intervention (n = 20) Week 12	Control (n = 18) Week 0	Control (n = 18) Week 12	*p*-Value ^1^
**Derived from arachidonic acid**					
**5-F_2_t-IsoP**	56.2 (±82.5)	39.2 (±37.3)	44.2 (±40.8)	63.3 (±40.8)	0.175
**15-F_2_t-IsoP**	31.8 (±37.1)	37.7 (±29.1)	51.7 (±62.1)	38 (±25.2)	**0.014**
**PGF_2a_**	109.1 (±128.7)	83.6 (±94.7)	112.2 (±122.6)	118.3 (±86.3)	0.217
**5-HETE**	179.9 (±158.8)	189.4 (±124.1)	197.4 (±130.8)	234.2 (±114.2)	0.675
**8-HETE**	67.6 (±71.3)	51.9 (±57.1)	74.8 (±46.3)	64.1 (±36.3)	0.734
**9-HETE**	178.3 (±161.8)	153.3 (±138.7)	166.3 (±95.0)	222.4 (±192.2)	0.069
**11-HETE**	96.5 (±86.8)	69.4 (±101.2)	131.1 (±98.6)	142.4 (±126.4)	0.119
**12-HETE**	112 (±52.9)	129.2 (±103.3)	150.5 (±64.1)	141.9 (±52.9)	0.303
**15-HETE**	139 (±106.8)	143.8 (±95.3)	153.5 (±77.3)	170 (±84.1)	0.696
**20-HETE**	337.1 (±240.6)	263.5 (±147.9)	375.7 (±218.2)	299.8 (±178.3)	0.588
**Derived from docosahexaenoic acid**					
**4-F_4_-NeuroP**	3890 (±3880)	4785 (±4415)	5388 (±5202)	4701 (±4322)	0.339
**4-HDHA**	349 (±246.8)	462.2 (±512.8)	368.9 (±390.0)	374.1 (±345.4)	0.696
**7-HDHA**	245.6 (±226.5)	303.9 (±288.8)	262.7 (±300.1)	279.6 (±403.6)	0.812
**11-HDHA**	704.6 (±938.6)	950.6 (±1625.0)	576.7 (±458.6)	743.1 (±666.3)	0.217
**14-HDHA**	4155 (±5985)	6220 (±12320)	4644 (±6696)	5349 (±6390)	0.800
**17-HDHA**	2205 (±1289)	3355 (±3557)	2734 (±2452)	2470 (±1918)	0.426

HDHA, hydroxydocosahexanenoic acids; HETE, hydroxyeicosatetraenoic acids; IsoP, isoprostanoids; PGF, prostaglandins; 1—*p*-values comparing fold changes in intervention and control groups during the 12-week intervention using Mann–Whitney’s U-test; bold marks significant values.

## Data Availability

The data presented in this study are available upon request from the corresponding author. The data are not publicly available due to ethical restrictions.

## Supplementary Materials

The following supporting information can be downloaded at: https://www.mdpi.com/article/10.3390/ijms24108509/s1. [89,90].
