# Supplementary material for: Twelve Weeks of High-Intensity Interval Training Alters Adipose Tissue Gene Expression but Not Oxylipin Levels in People with Non-Alcoholic Fatty Liver Disease
Source: Int J Mol Sci. 2023 May 9;24(10):8509. doi: 10.3390/ijms24108509 (PMC10218057; doi:10.3390/ijms24108509)
Supplement: Supplementary file 1 [file ijms-24-08509-s001.zip › ijms-2341638-supplementary/Supplements IJMS/Supplements_paper 3_completed.pdf]

Supplementary information

Figure S1. MSD plot of all 19 subjects with 2 AT samples at baseline and endpoint

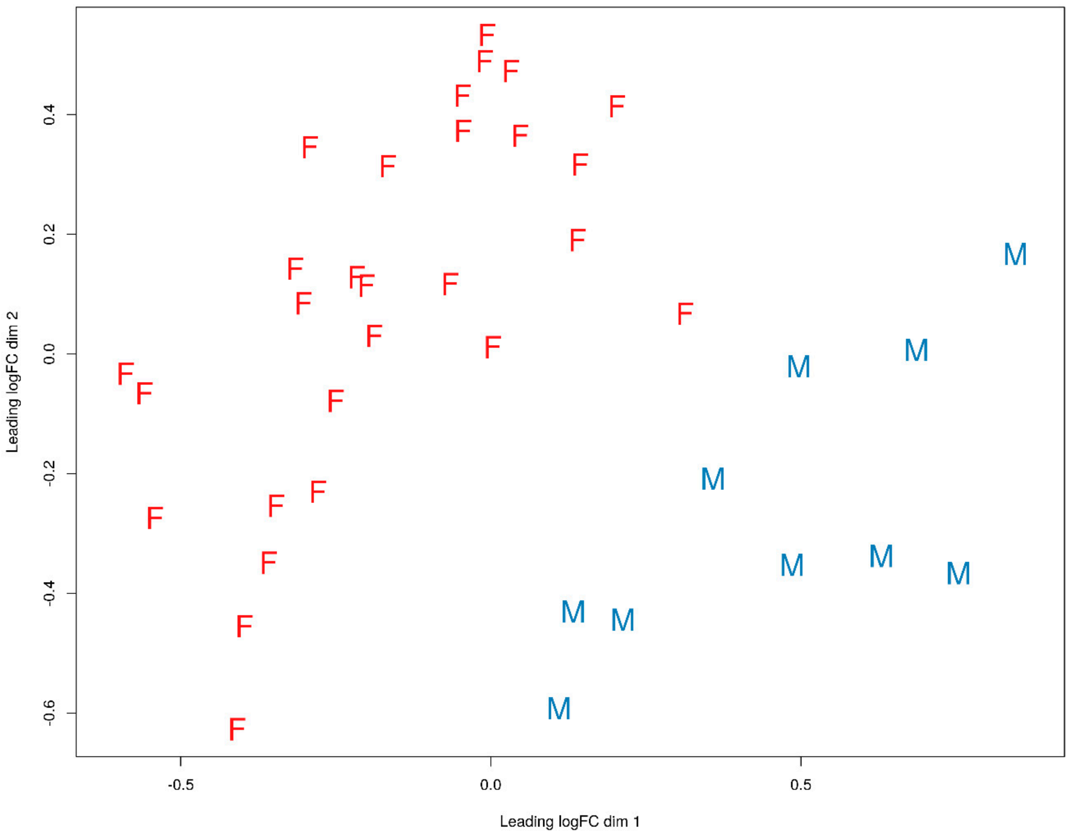

F female, M male

Table S1. List of genes with raw p-values and log2FC (fold change) comparing timepoints within the intervention and comparing intervention vs control during the intervention. (see extra excel file)

Figure S2. Heatmaps of SCD genes expression and BMI, waist, fat mass, visceral fat area, IHL (clustered based on SCD gene expression levels), gene expression shown as z-score

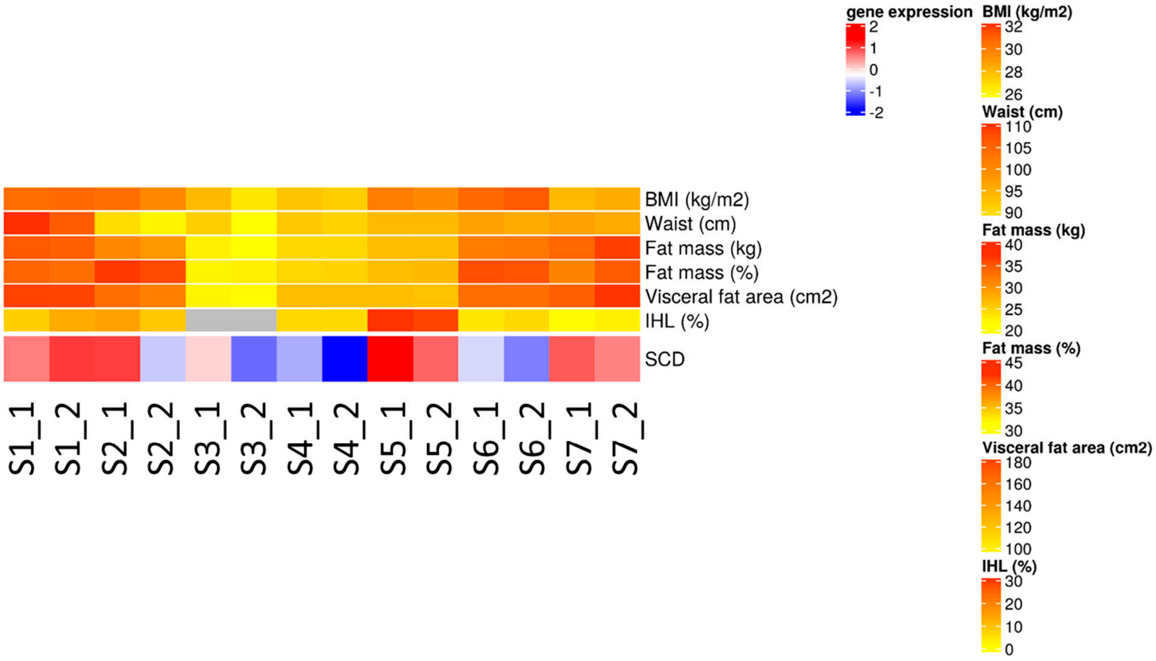

SCD Stearoyl-CoA Desaturase, BMI body mass index, IHL intrahepatic lipid content, S1_1 = sample 1 baseline (week0), S1_2 = sample 1 at endpoint (week 12)

Table S2. Fatty acid measured as fatty acid methyl esters (µg/ml) (FAME).

Fatty acid	I week 0	I week 12	C week 0	C week 12	p-value[1]
Palmitic acid	1351 (± 401)	1298 (± 503)	1109 (± 352)	1140 (± 412)	0.443
Palmitoleic acid	116.2 (± 49.4)	117.0 (± 52.1)	91.0 (± 44.2)	133.0 (± 125.8)	0.206
Stearic acid	617.2 (± 185.8)	531.9 (± 201.3)	496.5 (± 210.1)	507.2 (± 309.0)	0.119
Oleic acid	1121 (± 353)	1052 (± 419)	945 (± 316)	1082 (± 490)	0.186
Linoleic acid	1123 (± 334)	1037 (± 415)	1001 (± 355)	1060 (± 466)	0.478
Dihomo-γ-linolenic acid	167.4 (± 72.9)	143.6 (± 64.5)	148.2 (± 79.9)	158.1 (± 98.3)	0.133
Alpha Lipoic acid	45.5 (± 19.6)	41.3 (± 23.2)	40.8 (± 13.2)	46.8 (± 21.8)	0.112
Arachidonic acid	659.9 (± 245.5)	578.1 (± 247.9)	592.8 (± 335.6)	641.9 (± 424.5)	0.317
Eicosapentaenoic aicd	98.8 (± 40.1)	92.6 (± 59.9)	95.1 (± 81.0)	104.8 (± 83.4)	0.264
Docosahexanoic acid	322.8 (± 138.9)	254.0 (± 137.7)	209.8 (± 97.6)	221.1 (± 98.3)	0.131

C control, I intervention, **1** p-values comparing fold changes of C and I during 12 weeks using Mann-Whitney's U-test

Method S3a: Fatty acid composition measurement:

Plasma samples were thawed on ice and centrifuged at 3000 x g at 4 °C for 5 min to remove precipitates. Afterward, 300 µL 1X PBS, 50 µL of 0.2 µg/µL internal standard (nonadecanoic acid, C19:0), 550 µL MeOH and 28.2 µL HCl were added to 200 µL plasma sample, and vortexed for 1min. Then, 500 µL 1 N KOH(MeOH) was added and incubated for 1h at 25 °C on a shaker for the alkane hydrolysis. The reaction was stopped by adding 500 µL 1 N HCl. Thereafter, liquid-liquid extraction was performed by adding 1 mL isooctane, vortexed for 15 sec, and centrifuged at 2000 x g for 5 min at 25 °C. The top layer was transferred to a new glass tube and dried under nitrogen at 37 °C. The liquid-liquid extraction steps were repeated to receive a good amount of top layer. The methyl ester derivatives were created by adding 3 mL hexane: diethyl ether (80:20, v/v), vortexed for 15 sec, and centrifuged at 2000 x g for 1 min at 25°C. Again, the top layer was transferred to an autosampler glass vial. The steps were repeated, and the samples were then dried under nitrogen at 37 °C. After complete dryness, the residue was resuspended with 100 µL isooctane in an autosampler glass vial with an insert. The fatty acid composition was measured with Agilent 7890B gas chromatography (Agilent, USA) and an Agilent 5977A mass spectrometer (Agilent, USA) (GC-MS) according to previous studies [89,90]. Briefly, the injection port was set to 240°C and the GC-MS temperature to 250°C. The mass spectrometer was set at the electron capture negative ionization (ECNI) mode. An SP-2560 column with helium as a carrier gas was used (flow rate 1ml/min).

Table S3. Retention time, mass ions, and the ionizing energy of the metabolites

	RT (min)	Q1 (m/z)	Q3 (m/z)	DP (eV)	CE (eV)	ISTD

5-F2t-IsoP	9.71	353.23	115.0403	-50	-25	15-F_{2t}-IsoP-d4
15-F2t-IsoP	9.95	353.23	193.12	-50	-25	15-F_{2t}-IsoP-d4
4-F4-NeuroP	9.95	377.23	101.02	-60	-30	4-F4-NeuroP-d_4
PGF2a	9.95	353.23	309.21	-50	-15	PGF2a -d_4
5-HETE	11.75	319.23	115.04	-50	-15	5-HETE-d_8
8-HETE	11.64	319.23	155.07	-50	-15	5-HETE-d_8
9-HETE	11.69	319.23	123.08	-50	-15	12-HETE-d_8
11-HETE	11.58	319.23	167.12	-50	-15	12-HETE-d_8
12-HETE	11.63	319.23	179.11	-50	-15	12-HETE-d_8
15-HETE	11.55	319.23	219.13	-50	-15	12-HETE-d_8
20-HETE	11.43	319.23	275.24	-50	-15	20-HETE-d_6
4-HDHA	11.82	343.23	101.02	-60	-20	DHA-d_5
7-HDHA	11.70	343.23	141.06	-60	-20	DHA-d_5
11-HDHA	11.67	343.23	121.1	-60	-20	DHA-d_5
14-HDHA	11.61	343.23	205.12	-60	-20	DHA-d_5
17-HDHA	11.55	343.23	245.15	-60	-20	DHA-d_5
**Internal Standards**						
15-F_{2t}-IsoP-d4	9.92	357.26	313.23	-50	-25	-
4-F4-NeuroP-d_4	10.04	381.53	101.0246	-60	-30	-
PGF2a -d_4	9.92	357.26	313.23	-50	-15	-
5-HETE-d_8	11.73	327.28	116.0416	-50	-15	-
12-HETE-d_8	11.61	327.28	184.14	-50	-15	-
20-HETE-d_6	11.43	325.27	281.27	-50	-15	-
DHA-d_5		332.26	288.27	-50	-25	-

RT retention time, m mass, z charge number of ions, DP de-clustering potential, CE collision energy, ISTD internal standard spiking solution,
